# Adaptive compliant structures for flow regulation

**DOI:** 10.1098/rspa.2017.0334

**Published:** 2017-08-16

**Authors:** Gaetano Arena, Rainer M. J. Groh, Alex Brinkmeyer, Raf Theunissen, Paul M. Weaver, Alberto Pirrera

**Affiliations:** 1 Bristol Composites Institute (ACCIS), Department of Aerospace Engineering, University of Bristol, Bristol BS8 1TR, UK; 2 Department of Aerospace Engineering, University of Bristol, Bristol BS8 1TR, UK

**Keywords:** buckling, post-buckling, multistability, air inlet, adaptive structures, morphing

## Abstract

This paper introduces conceptual design principles for a novel class of adaptive structures that provide both flow regulation and control. While of general applicability, these design principles, which revolve around the idea of using the instabilities and elastically nonlinear behaviour of post-buckled panels, are exemplified through a case study: the design of a shape-adaptive air inlet. The inlet comprises a deformable post-buckled member that changes shape depending on the pressure field applied by the surrounding fluid, thereby regulating the inlet aperture. By tailoring the stress field in the post-buckled state and the geometry of the initial, stress-free configuration, the deformable section can snap through to close or open the inlet completely. Owing to its inherent ability to change shape in response to external stimuli—i.e. the aerodynamic loads imposed by different operating conditions—the inlet does not have to rely on linkages and mechanisms for actuation, unlike conventional flow-controlling devices.

## 1. Introduction

Engineering systems are generally designed to meet multiple requirements that derive from (i) the functionalities that a system is meant to fulfil and (ii) the expected operating conditions/environment. When viewed in isolation, individual requirements can drive designs in opposing directions.

The goal of classical design philosophies is to find the best compromise between competing drivers. The disadvantage of such design philosophies is that a system’s performance will be suboptimal in most, if not all, of the individual operating conditions. In structural engineering, one possible refinement to this traditional design approach is the use of the so-called morphing and adaptive technologies, which allow structures to change geometry and/or material properties in response to external stimuli [1]. Specifically, morphing and adaptive structures promise to enable less stringent trade-offs between stiffness, strength, weight and functionality [2–5]. Particularly attractive from a weight and minimal design philosophy perspective are passively actuated adaptive structures that do not rely on separate actuation devices to reconfigure their geometry [6–8].

The vast majority of the research efforts on shape-changing technologies take their inspiration from nature, where many organisms use compliance to adapt to changing environmental or operating conditions [9–11]. The adaptive aerodynamic shape of bird wings, for instance, is used in aerospace engineering as *the* archetypical example of evolution-optimized morphing.

**Figure 1.**
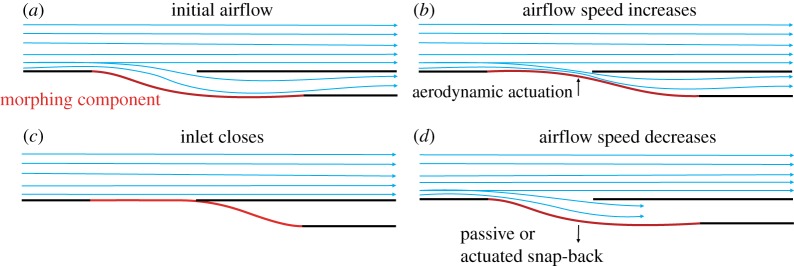
Schematic of adaptive air inlet. (*a*) The air inlet, with the morphing component (in red) in its open configuration, can be actuated (*b*) and closed (*c*) by the pressure field imposed by a fluid flowing at a certain speed over the curved structure. The multistable properties of the structure and the fluid’s boundary conditions at the end of the duct dictate whether the inlet remains closed or opens again when the air speed reduces (*d*).

In this paper, we present a novel design concept for an adaptive, variable geometry air inlet for flow control and regulation. The underlying working principle relies on the structurally nonlinear characteristics of a post-buckled beam. Figure 1 shows the design concept schematically.

The inlet comprises a deformable insert, set between rigid components, and a cover. These elements are arranged to form a channel that diverts part of the external flow to an outlet downstream. The deformable component morphs in response to the pressure field caused by the fluid flow. In particular, increasing air speeds create areas of low pressure that actuate the deformable component towards the cover, thereby closing the inlet. The morphing air inlet can therefore snap back and forth between an ‘open’ and ‘closed’ configuration, purely in response to air flowing at different speeds over the curved geometry. The kinematic characteristics of the shape adaptation depend on the nonlinear structural mechanics of the post-buckled member. In this study, we identify a taxonomy of nonlinear post-buckling behaviours and demonstrate their use for the design of adaptive air ducts.

A review of the relevant literature shows several examples of adaptive structures in which repeatable shape changes are obtained by either tailoring the properties [12–19] of the constituent materials or designing specific stress fields into a structure [20–25]. Examples of the former include the work by Santer [12] and Williams *et al.* [13]. Santer [12] demonstrated that viscoelasticity can temporarily lead to a loss of bistability and therefore cause an inverted dome to snap back to its undeformed state. Williams *et al.* [13] designed and characterized an adaptive vibration absorber with tunable natural frequency by exploiting the temperature-dependent elastic modulus of shape memory alloys. With regard to the latter, i.e. morphing through stress fields, Daynes *et al.* [24,25], for instance, manufactured a flap and an air inlet capable of snapping between two stable configurations. Both devices use prestressed composite elements for changing shape: the first [24] by locking in self-equilibrated stress fields during the manufacturing process and the second [25] by simple mechanical compression.

Shape adaptation has also been obtained via elastic instabilities and stiffness adaptation. On the subject of stiffness adaptation, Runkel *et al.* [26] report a thin-walled composite wing box that twists upon aerodynamic bending only past a prescribed load. The phenomenon is induced by means of a variable (load-dependent) torsional stiffness. In particular, ‘on-demand’ twisting of the beam structure is enabled by the onset of non-catastrophic buckling in one of the webs of the box section.

A common thread can be found among many of the above-mentioned works. Independent of the design features generating the capability of shape-changing, adaptation is realized through structural instabilities. This feature corroborates the fundamental idea behind the work presented in this paper: elastic instabilities or, in other words, the temporary loss of stiffness should no longer be considered as a catastrophic aberration [27], but rather exploited as a means for adaptation and multifunctionality [20–23,28]. With this new design paradigm, it follows that it would be possible to take advantage of concepts such as multistability and elastic ‘snap-through’ instabilities [29] to create repeatable, ‘well-behaved’ shape changes.

**Figure 2.**
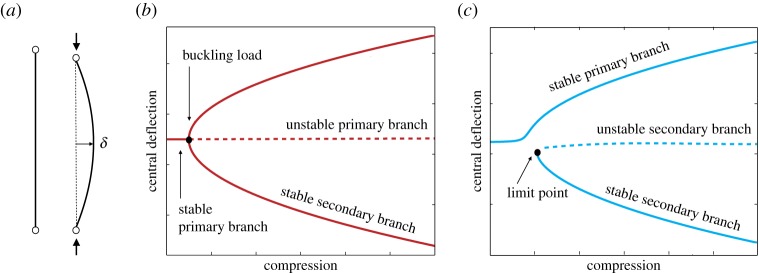
Buckling failure of a pin-jointed beam and corresponding bifurcation diagrams. (*a*) A pin-jointed beam bows sideways when subjected to a compressive force greater than the buckling load. (*b*) An idealized symmetric beam with no geometric or loading imperfections features a symmetric pitchfork bifurcation diagram in load versus displacement space. For small levels of compression the beam remains straight. This equilibrium destabilizes at the buckling load and the structure deflects into one of two mirror-symmetric configurations. (*c*) Conversely, the bifurcation graph related to a beam with symmetry-breaking geometry and/or loading is characterized by a ‘broken pitchfork’. It shows a primary stable branch and a secondary equilibrium branch. By applying a compressive load only, the structure naturally follows the primary branch, whereas the second configuration can only be reached by application of a transverse force.

Throughout this paper, we explore and then exploit buckling ‘failure’ for the design of the adaptive air inlet. In engineering parlance, the term buckling refers to a symmetry-breaking bifurcation, whereby a particular structural equilibrium (or state—often referred to as the fundamental state) becomes unstable causing a static or dynamic transition to a secondary configuration [30]. A typical example of buckling instability is the sudden loss of stiffness of elastic structures loaded in compression, as shown by the pin-jointed beam in figure 2. If the structure is designed such that the loss of stiffness, and therefore stability, is temporary such that load-bearing capabilities are restored before irreversible deformations occur, then buckling can no longer be considered as a failure mode, but rather as a means for shape adaptation.

## 2. Results

### (a) Buckling and post-buckling for shape adaptation

Figure 2 shows the idealized equilibrium curves for a pin-jointed beam subjected to axial compression. The curves are shown in terms of transverse deflection of the midpoint of the beam, *δ*, versus compression under load. Owing to its shape, this equilibrium manifold is often referred to as a ‘pitchfork’ bifurcation. For low levels of compression, the beam remains flat, but when the critical buckling load is reached, the primary flat state becomes unstable and the structure transitions into one of two mirror-symmetric sinusoidal modes, depending on small initial imperfections in geometry and/or load (figure 2*b*). In fact, moving away from this idealized scenario, any asymmetry in the geometry and/or loading conditions leads to asymmetries in the equilibrium manifold and ‘breaks’ the pitchfork, as shown in figure 2*c*. A broken pitchfork is characterized by a primary stable branch and a separate secondary branch with a ‘limit point’, implying that equilibrium can either be stable or unstable depending on the magnitude of the central deflection [29]. The secondary equilibrium branch cannot be reached by increasing the compressive load from the flat unloaded configuration. However, application of an additional transverse force, *F*, causes the structure to move from the stable primary branch to the stable secondary branch, traversing the solution manifold vertically and ‘snapping through’ a region of instability.

A post-buckled structure is said to be multistable when it can take two or more equilibrium states. As stable equilibria represent minima in the elastic potential energy landscape, they are always separated by a maximum, i.e. an unstable equilibrium. The presence of an unstable equilibrium between stable states implies that a loading case must exist for which multistable structures exhibit a dynamic ‘snap-through’ behaviour, when morphing from one stable buckled state to the other. Whether snap through is observed under a displacement-controlled or load-controlled transition between stable states depends on the particular shape of the load–displacement manifold. The reader is referred to [29] for further details on the fundamentals of elastic stability.

### (b) Multistability and snap through

**Figure 3.**
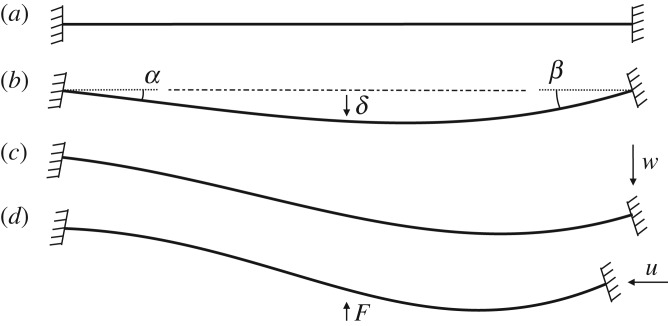
Parameters governing the elastic stability of a representative post-buckled beam. The buckling and post-buckling behaviour of a clamped–clamped beam (*a*) was studied by investigating the effects on elastic stability of different boundary conditions parametrically. In particular, we varied the beam angle at the extremities (*b*), the vertical displacement of one end (*c*), the compressive shortening (*d*), and the variation of stiffness (thickness) over the length. The beam is then snapped to the other side via vertical point force.

To develop the insight required for the design of the morphing member of the inlet, the geometrically nonlinear elasticity of a representative post-buckled clamped beam is investigated parametrically, as shown in figure 3. The results obtained permit a general understanding of how preloading and boundary conditions affect the post-buckling behaviour and its relationship with multistability. Specifically, with reference to figure 3 again, we studied the influence of parameters such as end rotations (*α* and *β*), end transverse displacement (*w*), compressive displacement (*u*) and thickness variation along the length of the beam (obtained by linearly increasing the thickness towards the right end). Each displacement condition—*α*, *β*, *w* and *u*—is applied sequentially, as illustrated in figure 3 with values as per table 1. The structure is then snapped into its inverted shape by means of a force-controlled arc-length method using finite-element code Abaqus [31]. For further details about the methodology, the reader is referred to §4.

**Table 1.** Sets of values used for the structural boundary conditions in the parametric analyses.

*w*	*α*	*β*	diameter
(mm)	(deg)	(deg)	(mm)
{1,2.5,5,7.5,10}	{0,−1.25,−2.5,−3.75,−5}	{0,1.25,2.5,3.75,5}	{3,lin. var.}

**Figure 4.**
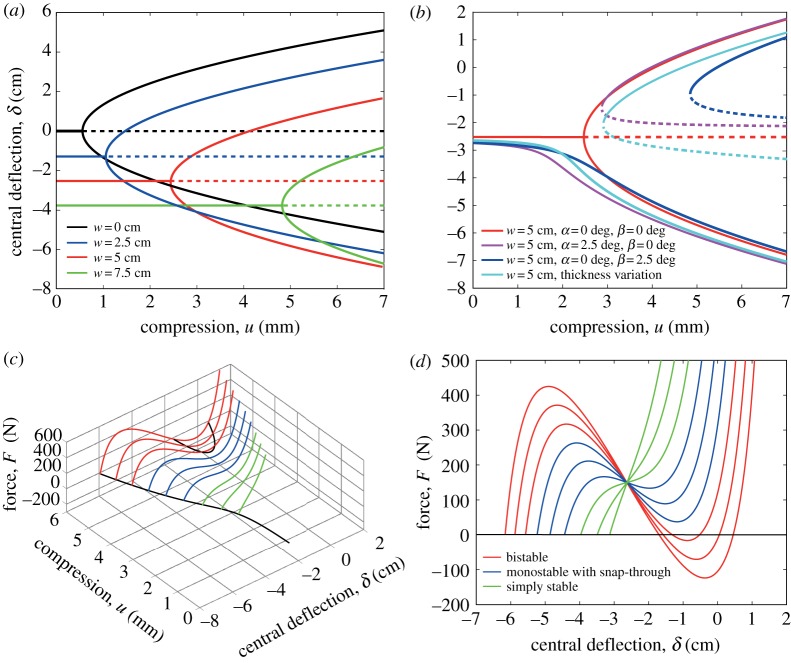
Bifurcation and force–deflection diagrams. (*a*) Bifurcation diagrams showing that imposition of a transverse displacement, *w*, at one end does not break the symmetry of the pitchfork equilibrium manifolds, but increases the compression at which buckling occurs. (*b*) End rotations, *α* and *β*, and thickness variation along the beam length break the equilibrium pitchforks by breaking the symmetry of the structure. Two separate equilibrium branches appear: A primary stable branch and a secondary branch with a limit point, corresponding to a change of stability. The application of *β* produces a larger separation between the limit point and the primary stable branch than the application of *α* or stiffness variations. (*c*) The relationship between buckling and multistability via a two-dimensional equilibrium manifold in compression versus central deflection versus central transverse force space. The equilibrium cut-sets in force versus deflection space demonstrate that, depending on the value of compression *u*, three different scenarios are possible: if the compression is greater than the limit point on the secondary equilibrium branch, the structure snaps through to a second stable state (red curves); by decreasing the compression, the structure is monostable but still exhibits snap through (blue curves); at a certain point snap through is no longer possible and the structure is simply stable (green curves). (*d*) An orthographic projection of the equilibrium manifold in force-central deflection space.

Figure 4 summarizes key findings from the parametric analysis. Figure 4*a* shows a cut-set of the equilibrium manifold in central deflection versus compression space, for different amounts of vertical displacement of the representative beam’s right end. All equilibrium loci are symmetric unbroken pitchforks, as suggested by the fact that application of *w* does not break the longitudinal (that is along the curvilinear domain) symmetry of the structure’s displacement field. The greater the vertical displacement, the greater is the required compressive displacement to buckle the beam.

For a given *w*, figure 4*b* shows the influence of end rotation (*α* and *β*) and the effect of longitudinal thickness variation on the equilibrium manifold. The rotational and thickness parameters introduce asymmetries that break the symmetric pitchfork, thereby resulting in two disconnected equilibrium branches. The specific shape of the broken pitchfork depends on the combination of the parameters’ variations. For example, the rotation *β* at the right end of the beam breaks the pitchfork and produces a large distance between the primary stable branch and the limit point on the secondary branch. Comparatively, this separation is smaller when an angle *α* is applied or the beam thickness is varied along the length. The degree of separation of the equilibrium branches is in fact crucial for design purposes, because the portion of the curve on the primary branch before the limit point of the secondary branch corresponds to a region of ‘monostability with snap-through behaviour’, which is of particular interest for the inlet design.

The findings of the parametric post-buckling study provide general guidelines for designing the adaptive air inlet. Figure 4*c* shows the relationship between buckling and multistability by means of a two-dimensional equilibrium manifold presented in compression versus central deflection versus central transverse force space. This solution manifold therefore superimposes the load–deflection curves of transverse force, *F*, versus central deflection on the bifurcation pitchfork diagrams, denoting the snap-through behaviour from one post-buckling configuration to another. The individual load–deflection snap-through curves are shown by means of an orthographic projection in figure 4*d*, where three distinct types of post-buckling behaviours are observed:

(i) For values of compression greater than the limit point on the secondary equilibrium branch, the structure shows the typical snap-through curves that intersect the central displacement axis at three points. These curves correspond to configurations where, when *F* is applied, the structure snaps from its first stable shape to its second configuration, which is stable even when *F* is removed.
(ii) For smaller compressions, the beam becomes monostable but exhibits snap-through behaviour when subjected to *F*. In this case, the structure does not have a second stable configuration when the external force is removed. Instead the system snaps back to its primary state upon load removal.
(iii) By decreasing the level of compression even further, the structure deforms nonlinearly displaying stiffness adaptation, but not featuring any snap through. In other words, the structure is simply stable.

In conclusion, the multistability of a clamped–clamped beam can be predicted in a straightforward manner simply by inspecting the bifurcation pitchfork diagrams. Therefore, to design an adaptive air inlet for a specific application and operational envelope, it is sufficient to produce and then study the equilibrium manifold of the morphing member. In the case of a symmetric structure, the post-buckled beam can only act in a bistable manner. When at least one symmetry is broken, the structure may also exhibit snapping monostability. The size of the region where monostability is observed can be controlled by changing the boundary conditions, as detailed in figure 4.

**Figure 5.**
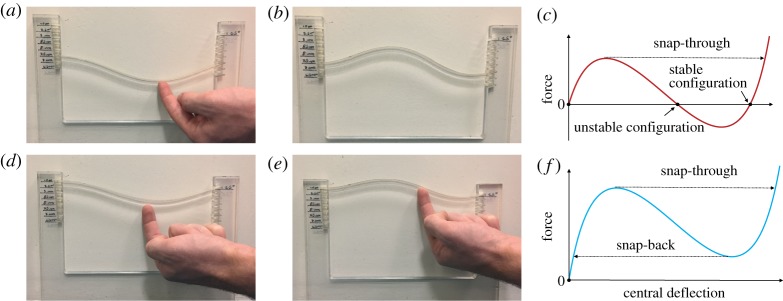
Multistability and snap-through behaviour. (*a*) The application of a transverse load to a bistable clamped strip in its first stable configuration causes snap through into the inverted stable shape (*b*). (*c*) The applied load increases until it reaches a critical value. At this point, the beam snaps through a region of instability, where applied load decreases, reaching a second stable branch. Upon load removal, the structures settles on the secondary stable state. Similarly, a monostable buckled structure snaps from its first (*d*) to its second inverted configuration (*e*) when a transverse load is applied, but, as shown in *f*, load removal causes snap back to the original unloaded equilibrium (*d*).

Multistability and dynamic snap-through instabilities are demonstrated by means of a toy model, as shown in figure 5 and electronic supplementary material, videos S1 and S2. Figure 5*a*,*b*,*d*,*e* shows the post-buckled configurations of a clamped–clamped strut with different boundary conditions. The stable states of the strut in figure 5*a* are connected by the equilibrium branch in figure 5*c*, demonstrating bistability. Similarly, figure 5*f* depicts the load–displacement diagram for the strut in figure 5*d*, demonstrating a monostable behaviour with snap-through instability. An immediate physical consequence of this difference in behaviour is that, although similar in shape, the structural configuration in figure 5*b* is in a state of stable equilibrium, whereas that in figure 5*e* must be held into position.

In summary, depending on pre-compression and boundary conditions, a post-buckled, clamped–clamped, slender panel presents the following taxonomy of nonlinear behaviours (see figure 4 for reference):

#### (i) Simple stability

The panel deforms nonlinearly when subjected to transverse load. No instabilities are observed. For increasing values of precompression, the load–displacement diagram transitions from a linear curve to one showing a softening–stiffening behaviour.

#### (ii) Monostability with snap through

A region of instability under load control is observed in the load–displacement diagram. This region is confined between a local maximum and a local minimum. The panel is able to snap through and so reach a distant inverted configuration. The inverted state is a stable equilibrium only under application of an external transverse load. The structure snaps back to the initial configuration when the transverse load is removed, because the load–displacement curve does not intersect the displacement axis other than at the origin (see figure 5*f* for reference).

#### (iii) Bistability

The panel exhibits two stable states upon application of a transverse load. When the applied force reaches a critical value, the structure snaps from the first stable state into the second configuration, traversing a region of instability. When the transverse load is removed, the structure remains in the inverted position at the point where the load–displacement curve intersects the displacement axis. Hence, a second stable unloaded configuration has been found (see figure 5*c* for reference).

This taxonomy of behaviours can be used to design adaptive inlets with different operational envelopes. Let us focus on the inlet in figure 1 and assume that it is designed to be fully open at low air speeds. An increase of fluid velocity generates an area of low static pressure over the adaptive component. This pressure field is equivalent to a transverse load. A morphing inlet with simple stability would thus deform to decrease the inlet aperture. In this case, the extent of flow regulation is proportional to air speed and limited by the stiffness and maximum displacement of the adaptive member. Bistable or monostable inlets with snap-through behaviour can be designed to have the aperture decrease for increasing air speeds and close completely upon snap through, when a critical speed is reached. In this respect, these two designs are similar, but they feature a fundamental difference for reducing air speeds. In particular, monostable inlets are able to snap back to the open configuration autonomously. Conversely, bistable ones do not need external inputs to hold the closed, inverted shape. As a consequence, they only require actuation to snap open. In conclusion, a monostable inlet with snap-through behaviour is a completely autonomous adaptive system. Similarly, a bistable one does not need continuous actuation to close, but it does require an external input to reopen.

### (c) Adaptive air inlet

**Figure 6.**
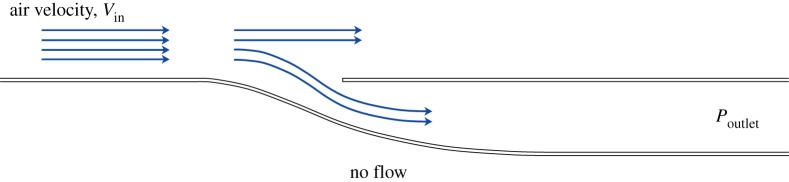
A portion of the computational domain for the fluid–structure interaction model. Air flows from left to right. Pressure boundary conditions are applied at the outlet, together with a no-flow condition below the structure.

The parametric buckling and post-buckling study outlined in previous sections provides the insight required to design bistable or monostable beams. This insight is used to design the deformable portion of the adaptive air inlet shown in figure 6—one with bistable and one with monostable characteristics.

**Figure 7.**
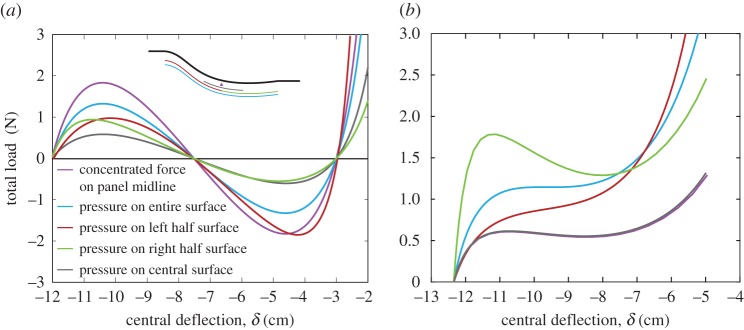
Snap-through behaviour of bistable and monostable adaptive inlets. (*a*) The force versus central deflection of the bistable inlet—obtained by applying a compressive load greater than the buckling load and maintaining the symmetry of the structure—intersects the central deflection axis three times, indicating bistability. (*b*) Breaking the axial symmetry of the structure by varying thickness along the length results in monostable snap-through behaviour.

Figure 7 shows the force–displacement curves for these bistable (figure 7*a*) and monostable (figure 7*b*) devices, which match the characteristics of the curves depicted in figure 5. Both structures exhibit snap-through behaviour, but with different loading–unloading paths. As desired, the bistable structure has a second stable configuration in its unloaded state, whereas the monostable beam snaps back upon removal of the transverse load. Figure 7 compares the snap-through behaviour of the bistable (figure 7*a*) and monostable (figure 7*b*) structures in reaction to a point force at the central nodes of the arch and a uniformly distributed transverse load across the entire, left half, right half and middle upper surface of the structure. For both the bistable and monostable cases, the type of loading affects the snap-through behaviour quantitatively in that the total load acting on the beam at snap-through changes. On the other hand, stability characteristics remain unchanged regardless of whether the load is distributed or not and in which region it is applied.

**Figure 8.**
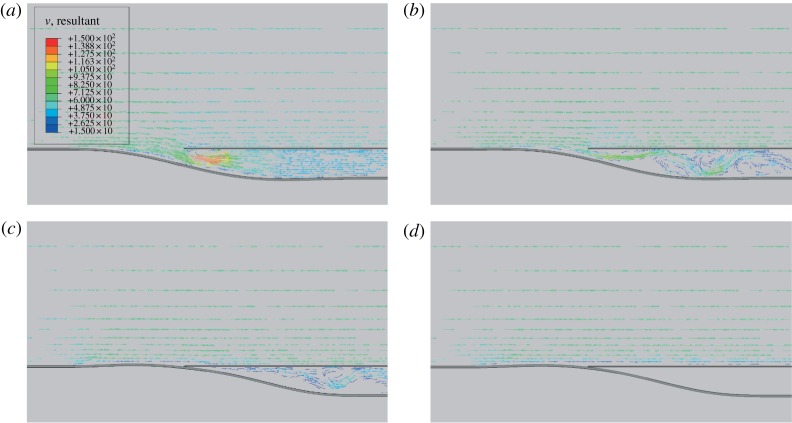
(*a*–*d*) Passive actuation of a bistable adaptive air inlet. A 60 m *s*^−1^ air flow above the bistable inlet causes a pressure field that actuates snap through from the initially open state to the closed state. The bistable configuration holds its closed configuration even when the air flow ceases due to its structural characteristics. The monostable inlet (not shown) shows similar behaviour, but the closed configuration is not stable with respect to decreasing air speeds. Coloured arrows represent the velocity vector field, with minimum and maximum magnitude speeds indicated in blue and red, respectively. From the open to closed state, the snap through takes of the order of 10 ms.

Both the bistable and monostable morphing inlets are immersed into a 60 m *s*^−1^ airflow, which is sufficiently fast to cause snap through. With reference to figure 6, a ‘no-flow’ condition is imposed below the structure. The gauge pressure at the outlet, i.e. at the end of the duct, is set to be lower than the static pressure of the external air. This fluid boundary condition is application-specific. Low downstream pressures are representative of applications in which the air diverted by the inlet joins faster and/or cooler flows. It is important to note that the outlet pressure is a fundamental parameter, which drives the fluid–structure interaction (FSI) and, therefore, influences the structural design of the adaptive component. The behaviour of the inlets when submerged in a fluid flow is shown in figure 8 and electronic supplementary material, videos S3 and S4. FSI simulations demonstrate that the airflow induces a pressure field over the inlet that actuates the deformable structure to snap into the second ‘closed’ configuration. The coloured vector contours in figure 8 indicate air velocity, with low speeds in blue and higher speeds in red. As expected, the closed state of the bistable inlet, shown in figure 8*d*, is stable even when the air ceases to flow. The monostable device exhibits a similar snapping response, but the inlet opens again at reduced air speeds, thus showing valve-like opening-and-closing behaviour. Preliminary observations suggest the existence of conditions under which high-frequency oscillations of opening and closing cycles can be induced. However, the stability of the closed state can also be controlled by the outlet pressure, meaning that, a specific outlet pressure can be prescribed to keep the monostable inlet in its closed state upon snap through.

## 3. Discussion

Compliant devices are increasingly being investigated by the scientific and engineering communities, due to their unique capabilities for shape adaptation in response to changing environmental or operating conditions. Nevertheless, many adaptive morphing apparatuses still rely on external actuation, be it mechanical, thermal or piezoelectric [1,15,32]—e.g. the bistable air inlet designed and manufactured by Daynes *et al.* [25]. A passively adaptive structure, which does not rely on external mechanisms for actuation, but rather responds to external stimuli to drive actuation, either by changing internal constitutive parameters or by exploiting fluctuating external forces, promises less stringent compromises between load-carrying capability and functionality, while shedding the additional mass of the external mechanism.

Historically, structural instabilities, and the multistability derived thereof, have been considered as catastrophic and detrimental phenomena [27]. Morphing technologies, on the other hand, have shown that structural instabilities can be an effective means for shape change [20–23] and have placed their utility into new relief. These concepts are investigated and exploited herein to design an adaptive air inlet that snaps between open and closed configurations depending on pressure conditions induced by air flowing over a morphing component.

The insight gained from an initial parametric study on the effect of different boundary conditions on the post-buckling behaviour of a clamped–clamped beam (figure 4) shows that multistability of a one-dimensional morphing component can be readily controlled by varying the end conditions, such as longitudinal compression, transverse displacements and rotations. Indeed, we have derived a taxonomy for nonlinear behaviours, demonstrating that the desired multistability and snap-through behaviour of a morphing component for a specific application can be evaluated simply by plotting and studying the equilibrium manifold of the structure. In particular, we have demonstrated, that by introducing asymmetries, it is possible to transition from the bistable behaviour of the classical elastica to a monostable design that still exhibits snap through and therefore the much desired large deformations. In deflection versus compression space, the specific region of monostability, situated between the limit point of the secondary stable branch and the primary equilibrium curve of a broken pitchfork, can be controlled by varying the parameters of the system, i.e. compression, end rotations and vertical end deflections. This insight is important for design purposes as it allows the design of both binary inlets, statically stable in both open and closed configurations, and valve-like inlets, which can passively transition between open and closed states. The FSI simulations presented in this paper shows that these two types of device can be actuated passively simply by exploiting the changes in the pressure field caused by air flowing over the curved inlet (figure 8). For the particular example studied herein, snap through is activated at a velocity of 60 m *s*^−1^, at which point the inlet closes within a time frame of approximatively 10 ms so that air can no longer flow into the duct.

The actuating flow speed can be tailored to specific applications by varying parameters such as material properties and structural boundary conditions. The appropriate design can be chosen by conducting a parametric post-buckling analysis and studying the resulting equilibrium manifolds.

In conclusion, this paper introduces a new framework for exploiting structural instabilities as an engineering design tool. The multistability of simple one-dimensional beam structures is used to design a passively actuated variable geometry air inlet. The passive actuation mechanism renders the proposed concept as a lightweight solution that is not subject to weight and volumetric restrictions. As a result, the proposed morphing air inlet promises to benefit many applications where FSIs and shape adaptation are key, e.g. in the biomedical, automotive and aerospace industries.

## 4. Methods

All simulations are performed in Abaqus Unified fea [31], a Finite Element software suite by Dassault Systèmes Simulia Corp.

### (a) Parametric nonlinear beam structural analysis

The design of the adaptive air inlet is based on the parametric study of the buckling and post-buckling behaviour of a representative one-dimensional beam of unit length and circular cross section. The parameters used in this investigation are presented in table 1, with their physical meaning illustrated in figure 3. Structural analyses have been run for all possible combinations of the values indicated in the sets. Equilibrium manifolds in transverse central deflection versus compression space are traced numerically by means of the Abaqus implementation of the arc-length method based on Riks’ formulation [33]. Load–displacement curves are traced using the same formulation with load as the arc-length parameter. In particular, a transverse force is applied at the midpoint of the beam to make the structure transition towards the inverted equilibrium state. These calculations are performed for three different values of compression, *u*={1,3,6} mm. For the finite-element simulations, the beam is discretized with 2-node linear beam elements of the type B31. A total of 140 elements ensures a converged mesh.

### (b) Inlet geometry, materials and structural analysis

**Figure 9.**
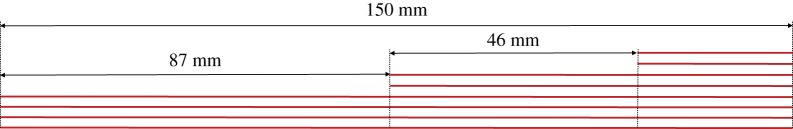
Composite lay-up for the monostable inlet with snap-through behaviour. Red lines represent composite layers. Step changes in thickness cause stiffness variations and the structural asymmetry required for monostable behaviour with snap through.

The material of choice for the adaptive part of the inlet is unidirectional glass fibre epoxy resin composite, Glass/913 (Material properties as shown in table 2). An isotropic elastic material with Young’s modulus of 70 GPa is used for the inlet cover and rigid components. The composite member measures 150 mm in length and 11.2 mm in width. The bistable design is composed of eight layers of glass fibre for a total thickness of 1.04 mm. A 15 mm vertical displacement and a 1.5 mm compression are applied to one end of the inlet, with no rotations at either side. The monostable design is derived from the bistable one altering geometry and preload. In particular, the thickness is varied along the beam length as shown in figure 9 and the longitudinal compression is lowered to 1.3 mm. All other properties and boundary conditions are kept unchanged. As a preliminary study, the nonlinear snap-through behaviour in the absence of air is evaluated, again using an arc-length Riks algorithm [33]. For both the mono- and bi-stable structures, an 8-node linear brick element (type C3RD8R), with reduced integration and enhanced hourglass control, is selected. A fine mesh with 568 elements is used along the beam length, which is sufficient to ensure convergence of the nonlinear post-buckling behaviour. One element for every two layers is used in the thickness direction; further fidelity is not necessary because the composite lay-up is unidirectional.

**Table 2.** Glass/913 material properties.

*E*_1_	*E*_2_	*G*_12_	*ν*_12_	thickness
(GPa)	(GPa)	(GPa)	(-)	(mm)
43.7	7.5	4.3	0.3	0.130

### (c) Fluid–structure interaction

The effect of the aerodynamic loads on the adaptive air inlet is studied by means of FSI simulations. Owing to extreme deformations of both the structural and fluid domains, a Coupled Eulerian–Lagrangian (CEL) approach is chosen [31,34]. An explicit integration scheme is used, with an automatic, adaptive time step. Figure 6 shows a diagrammatic representation of the FSI model. The inlet is depicted in dark green. Numerically, the structure is represented by a Lagrangian orphan mesh imported from a structural-only analysis where the inlet is forced into its post-buckled state. In the FSI simulations, post-buckling stresses are applied as a predefined field. The Lagrangian inlet is then ‘immersed’ in a Eulerian domain completely filled with air to ensure a Eulerian Volume Fraction (EVF) of fluid consistently equal to 1 everywhere. The air volume is modelled as a Newtonian fluid with standard properties at 25 ^°^*C* and atmospheric pressure, i.e. density *ρ*=1.205 kg m^−3^ and viscosity *μ*=1.915×10^−5^ Pa⋅s [35]. CEL simulations require the speed of sound in air to be defined (*c*=346 *m* *s*^−1^ at 25 ^°^*C*) as a material input. This value is used to calculate elastic bulk modulus as *ρc*^2^, which is a measure of the fluid compressibility. A gauge relative pressure, *P*=0 Pa, is assigned beneath the air inlet where no fluid is assumed to flow. When using the CEL method, by default, the boundaries of the Eulerian domain with set pressure values reflect pressure waves [31], which affect the numerical solution adversely. This problem is avoided by assigning a uniform initial velocity field throughout the entire Eulerian domain. Additionally, a sufficiently long duct must be used and/or a negative gauge relative pressure, *P*_*out*_, needs to be imposed to avoid flow reversal. Negative values are used here to represent applications in which the duct connects to interfacing components that allow the air to naturally flow out. Both the bi- and monostable inlets are designed for a snap-through velocity of about 60 m *s*^−1^. This value is therefore imposed as the initial fluid velocity for the Eulerian mesh.

A default penalty contact method manages the solid–solid interaction, while the so-called volume of fluid (VOF) method [36] is used for the fluid–solid interface tracking. The VOF method enforces the contact constraints and no-slip conditions when, at the specific interface nodes, the arithmetic mean of the EVF of the surrounding elements is higher than 0.5 [31]. Only one element is used through the width of the domain (into the page) as three-dimensional effects are neglected. The size of the fluid domain is chosen to be large enough (0.5×0.4 m) to minimize boundary effects. For accuracy and convergence, the fluid mesh is refined homogeneously resulting in 700 000 8-node linear Eulerian brick element of type EC3D8R.

## Data accessibility

All underlying data to reproduce the results and support the conclusions are provided within this paper. Numerical models are available for download from the Research Data Repository of the University of Bristol at https://data.bris.ac.uk/data or directly via the doi:10.5523/bris.268qr4fvye59d2258ktk2ebhrp.
